# Wanting to or having to – a qualitative study of experiences and attitudes towards migrant screening for tuberculosis in Norway

**DOI:** 10.1186/s12889-019-7128-z

**Published:** 2019-06-21

**Authors:** Ingunn Nordstoga, Mona Drage, Tore Wælgaard Steen, Brita Askeland Winje

**Affiliations:** 1 LHL International Tuberculosis Foundation, Oslo, Norway; 2 Health Agency, City of Oslo, Norway; 3 Norwegian Institute of Public Health, Oslo, Norway

**Keywords:** Tuberculosis, Screening, Immigrants, Stigma, Health literacy, Health communication, Accessibility, Autonomy, Ambivalence

## Abstract

**Background:**

This study assesses how tuberculosis (TB) screening is perceived by immigrants in Norway. Screening is mandatory for people arriving from high incidence countries. To attend screening, immigrants have to contact the health system after receiving an invitation by letter. The proportion of non-attenders is not known, and there are no sanctions for not attending. Generally, only persons who test positive receive test results. The study explores users’ experiences, attitudes and motivations for attending or not attending TB screening, and perceived barriers and enablers.

**Methods:**

We conducted six focus group discussions and three individual interviews with 34 people from 16 countries in Africa, Asia and Europe. Interviews were recorded and transcribed, and data was coded following a general inductive approach: All transcribed text data was closely read through, salient themes were identified and categories were created and labelled. The data was read through several times and the category system was subsequently revised.

**Results:**

Most appreciated the opportunity to be tested for a severe disease and were generally positive towards the healthcare system. At the same time, many were uncomfortable with screening, particularly due to the fear and stigma attached to TB. All experienced practical problems related to language, information, and accessing facilities. Having to ask others for help made them feel dependent and vulnerable. Positive and negative attitudes simultaneously created ambivalence. Many wanted “structuring measures” like sanctions to help attendance. Many said that not receiving results left them feeling anxious.

**Conclusions:**

In order to adapt the system and improve trust and patient uptake, all aspects of the screening should be taken into account. Ambivalence towards screening probably has a negative impact on screening uptake and should be sought reduced. A combination of ambivalence and a wish for “structuring measures” leads the authors to conclude that mandatory screening is a reasonable measure. However, since mandatory screening negatively impacts patient autonomy, and because of fear, stigma and practical problems, the health system should empower users by improving communication and access to services. In addition, it is recommended that negative test results are also communicated to the users.

**Electronic supplementary material:**

The online version of this article (10.1186/s12889-019-7128-z) contains supplementary material, which is available to authorized users.

## Background

Tuberculosis (TB) screening for immigrants from high incidence to low-incidence countries is common but controversial. Issues that have been discussed include the yield of the screening programmes, cost-effectiveness [1, 2], and whether screening should be mandatory or voluntary [3, 4]. In studies focusing on user perspectives, issues related to accessibility have been raised and it has been questioned whether screening is discriminatory, stigmatising or racist [5] In order to obtain legitimacy and improve uptake, screening should be perceived as acceptable by its users. In this study, we assess how TB screening and the health system are perceived by immigrants in Norway. We explore motivations for attending or not attending the screening programme, as well as perceived barriers and enablers.

Norway is a country with low TB incidence, but with a high incidence in certain groups. About 90% of notified TB cases are found among foreign-born individuals. In 2017, 231 of 261 notified patients were born outside Norway; 109 came from Africa, 97 from Asia, 54 from Europe (including Norway) and one from South and Central America. Eritrea, Somalia, Philippines, Pakistan and Ethiopia were the most common countries of origin among the foreign born patients [6].

Norway has a well-established screening programme with mandatory screening for immigrants from countries with high TB incidence. The screening programme includes screening for both active TB and latent TB infection (LTBI) and typically includes symptom screening, chest X-ray and Interferon-Gamma Release Assay. LTBI screening is targeted at individuals younger than 35 years. At the time of the study, the definition of a high TB incidence country was more than 40 cases per 100,000 population. This has later been changed to 200 per 100,000, and also including people from Afghanistan and Eritrea because these two countries have high incidence rates in Norway [7]. The proportion of TB cases detected by arrival screening has increased over the last years. In the period 2015–2017, 22–40% of the cases were detected by arrival screening [6].

The number of arriving immigrants who are obligated to undergo screening and their reason for immigration varies over time. At the time of the study there were approximately 40,000 individuals eligible for screening per year and among them 31% were asylum seekers [8]. The screening pathways varies between the immigrant groups. Asylum seekers are screened at reception centres, while other immigrants are screened in the municipalities [9, 10]. The latter will receive a letter from the municipality after arrival informing them about the screening and requesting them to book an appointment. There is no standard format or content of the letter from the municipalities, but at a minimum the letter informs that the screening is mandatory for people from high TB incidence countries, and that the screening is free of cost. Individuals with indications of TB or LTBI should be referred to specialist health care for follow-up and treatment. Sending test results only to those who test positive is the common routine in most municipalities.

According to the regulations, screening should be performed as soon as possible after arrival in Norway, and latest within 14 days for asylum seekers and preferably within 4 weeks for other immigrants [11]. Although screening is mandatory, there are no sanctions for not attending. Non-attenders are not systematically registered, and the proportion of non-attenders is not known. It is assumed that most asylum seekers undergo screening at the reception centres, whereas the proportion screened in the municipalities is unknown and probably varies geographically and across groups. We assume the risk of non-attendance is higher among immigrants who have to book an appointment themselves. For this reason we focus on immigrants other than asylum seekers in this study.

The current monitoring and evaluation system of the TB screening programme is weak. Multiple service providers are involved in the screening process and there is no harmonization of data-collection or follow-up. Our study is among several initiatives to improve this. In order to explore users’ experiences and perceptions of the system we conducted a qualitative study. Qualitative studies are well suited to explore subjective meanings, actions and social contexts, as understood by research participants themselves [12].

## Methods

We conducted six focus group discussions and three individual interviews including in total 34 persons. One focus group and one individual interview were conducted in English, the others in Norwegian. Because Norwegian and English were not the participants’ native languages, questions were formulated as simply as possible and clarifications of questions and responses were put forward if necessary. Two participants in the Norwegian-speaking focus groups had very limited Norwegian abilities, so a translator for the native language was used. Participants were recruited through our contacts in the immigrant population, Oslo Adult Education and the Service Centre for Foreign Workers. Participation was based on written informed consent. Participants were able to choose the location of the interview. Focus group discussions and the majority of the interviews were conducted at the Oslo Adult Education offices or at the researchers’ office.

We collected information about country of origin, sex and reason for immigration. Participants came from 16 different countries in Africa (23), Asia (9) and Europe (2). Twenty-three were women and 11were men. Twenty-three came for family reunification, three were au pairs, two were work migrants, and six were students. Thirty-one had attended screening whilst three had not (For details see Additional file 1).

An interview guide was developed in Norwegian and English (Additional file 2). Open questions asking participants to tell about their experiences, attitudes and motivations for screening, as well as perceived barriers and enablers, were formulated. The questions regarded information given during the screening process, appointment-making, access, examinations, motivations for attending/not attending, and positive or negative experiences with the screening. They proceeded from simple questions about experiences to more complex questions about perceptions and attitudes. During focus group discussions and individual interviews, questions from the guide and questions generated on-site were asked. Discussions and interviews were audio-recorded and transcribed, and data was coded following a general inductive approach: All transcribed text data was closely read through and salient themes in the text data were identified. Key themes were identified, and from these, categories were created and labelled. The text data was read through multiple times and the category system was subsequently revised. A COREQ checklist is included (Additional file 3).

Because screening for TB and LTBI is closely linked in the screening programme and participants did not distinguish the two objectives of the screening, we have not differentiated between screening for TB and LTBI in the study.

## Results

### Attitudes towards screening and motivation for attending or not attending

Most participants stated they were positive towards TB screening. An important reason for this was prior experience of TB. Some had seen people very ill and even dying from the disease and hence felt reassured by getting tested. One said:

“During our flight from Somalia, we got to know people who had symptoms of TB and who died of it. (..) Based on that experience, I thought it was very good that one is given the opportunity to check if you have this disease.”

The fact that they had seen people being sick with TB made them perceive screening as a relevant and good thing: Many said they wanted to know if they were healthy. They also underlined that screening would give them the opportunity to get treatment if sick: “Everybody knows that if you don’t do anything about the illness today, you can be in danger tomorrow”. Many expressed they wanted to be responsible and not infect others if ill. Several mentioned that they knew of other people with TB who didn’t care about whether they infected others or not.

Some women saw the screening as a sign of care: One said: “When I got the letter”, I got the impression that somebody was concerned about my health. That was a good feeling. “I just came here, and somebody is caring for me already?”

An important reason for attending screening was to “follow the rules”: One said: “I was just following the rules in the country I was living in.”

A minority said they did not find it beneficial to be screened because they had been tested for TB or were BCG vaccinated in their home countries.

Two mentioned they had heard that there had been a TB epidemic in Norway previously, and asserted that this was the reason for screening. One claimed that TB was not at all a problem in his county, and stated that “Norway has had a history of TB. It should be said that this is why they test people for TB”.

For many, screening was performed a long time after arrival in Norway. Several said that if they had TB, they would have infected others during this period. One said: “I was here for six weeks before checking if I have it or not. So, 100 %, if I had it, I would have transferred it to anybody.”

Those who had not attended screening mentioned several contributing factors. One was pregnant at the time of arrival, and had little energy. She said she felt certain she did not have TB, and had a lot to think about during her initial period in Norway. After some time, she moved to a new municipality, and subsequently forgot about the screening. Another said she was preoccupied with getting settled and did not know how to attend screening: She understood the letter was about TB, but did not understand how to follow up: “My husband understood, but he was very busy, and said I had to book an appointment myself. But I don’t know how to do it.” Still, after more than 2 years in Norway, she did not know how to get screened or whom to ask. A male student, said: “I didn’t think it was necessary for me to go. I was busy doing another paper work, and looking for a job (…) and I had taken the vaccine before.”

### Fear and stigma and their relation to attitudes towards testing

Many participants talked about fear related to TB and how it affects attitudes and experiences of screening. One informant talked about the great fear she had of TB and compared it to the fear related to HIV: “For instance, with HIV, you just panic, no matter how much you trust yourself. (..) That kind of feeling comes. Maybe I have these things, even if I don’t know. So that feeling comes, of course, until the results come”. Another said she “freaked out for hours” when receiving a letter from the authorities some days after screening. She had been told she would be contacted if the tests were positive, and did not calm down until she got help with translation and understood the letter was about something else. Several found the screening setting uncomfortable, and one said going to the hospital is like going to the police: “The hospital is like, you know, police. You come to Norway, and all of a sudden, you have to go to the police. It is like the same.”

Contrary, others emphasised that they had never heard of people being afraid of TB or screening. One said: “I have never heard that people react in that way (being afraid). No, I have never heard that. I have only heard the complete opposite, that people want to take the test. (…) This is completely new thoughts for me”.

As regards to perceived stigma, it was common to talk about others who felt stigmatised, but few talked about feeling stigmatised themselves. One said: “In my country, we talk a lot about TB, but those who have TB don’t talk about it, because they feel ashamed”. Several referred to people who hid their TB diagnosis and how they believed this was bad.

For those who expressed that they had felt fear and stigma, important reasons were related to being singled out for testing: One said “It would be good if they explained the background for why a person is tested, and that it is not only him, (…) it is all people who come from that area. This would make the person feel safe about taking the tests, and not be afraid.” Several said that by not receiving this information, they felt stigmatised, fearful and as if regarded with suspicion.

Some expressed ambivalent attitudes. One said: “I did not experience it as something positive at all, because there is much stigma attached to the disease (…). But today, when I know what I know now, I think it is positive.” Others cycled between positive and negative thoughts. One said: “I only see the positive sides of it. They just want to protect people. That’s all.” Later in the interview she said: “(...) I don’t think it is comfortable at all (...) It makes you worry (…) Just the paper arriving is not by any means making things easy.”

### Perceptions of the screening information and the Norwegian health system

All were informed about the screening through an invitation letter, either in Norwegian only or in Norwegian and English. Nobody understood Norwegian at the time of receiving the letter and many did not understand English. One said: “I didn’t understand, so I just put it somewhere”. Many had spouses who translated it for them: “My husband translated. He is a teacher and understands, so it was easy. But for others it is difficult”. Having to ask others for translation of confidential letters made them feel insecure: “You don’t have any privacy. Other people know things about you. I have seen people going from one person to another. You have to find out whom you can trust.” Almost all underlined that it would be important to have information in a language they could understand. One said: “We don’t take it seriously when it is in Norwegian”. Another expressed that it was important that the letter was written in Norwegian in addition to their own language in order to confirm that the letter was in fact written by the authorities in Norway.

Regarding the content and “tone” of the letter, many stressed that a strict and even threatening tone was necessary. Several stated that many people are “lazy” and therefore should be informed that attendance is not only compulsory, but that there would be consequences for non-attendance: “People are lazy (...). There is nothing in the letter about punishment or anything”. One Somali participant said: “People from Somalia like that you talk like a soldier. They understand in this way.” Two suggested that attending screening should be a requirement for obtaining a residence permit. Those who stressed the necessity to be strict still appreciated friendliness from the health personnel.

Many said the rationale for screening was not well explained, and wanted more information: One said: “Naturally, I wouldn’t do something that I don’t understand.” They also wanted information about consequences of a positive test. However, it was important that this message was not designed to cause fear. Suggestions were: “You will be taken care of” and “You will not be sent back to your home country”.

Most participants were positive towards the Norwegian health system. They found health personnel friendly and well qualified and the health facilities well equipped. Several noted the difference from that of their home countries and felt that they would be better treated in Norway. Examples of statements were: “Health personnel have a lot of respect, for Norwegians and for others. That’s good.”, “I believe Norway is doing everything best” and “We (Somalis) like to be checked – many want to check their entire body. CT or MR; we want to be inside there to be checked, the whole body from bottom to top”. Some were positively surprised that screening was free, and stressed that in their home countries they would have had to pay a lot for this testing.

However, many perceived the system as slow and “diffuse” due to long waiting times and unclear messages. One said: “In Norway, there is a lot of “maybe””. It was difficult not to be told a fixed time for receiving results: One said: “They say “in about 2 weeks”. What does “in about 2 weeks” mean? (…) I don’t like “about”.” Most who had tested negative had not received results but had been told that no results was a sign of a negative test. Almost everybody said that they would prefer to receive results, even if negative, because without results being explicitly communicated it was difficult to feel reassured. One said that receiving results “is like peace of mind”.

### Practical aspects of screening, the situation of recent immigrants that influence health behaviour, enablers and disablers

Most participants had experienced practical problems during the screening process. Many said that since they were new in Norway, they did not understand the process. They were guided by spouses, other relatives or friends, if they understood and were willing to help, but felt very dependent on them: “I just followed my husband. (…) I did not understand the language. I had just arrived. My husband explained everything.”

Many had difficulties with booking appointments due to problems with language, not understanding the system or long waits on the phone. In addition, they were preoccupied with getting settled and it was difficult to remember to attend screening. For some finding transport, locating the facilities and travelling long distances to the screening facilities were inhibitory factors.: “If I was alone (husband followed), I would never have found it.” Many spent a lot of time searching: “When I got there, I already had walked for an hour looking for the place (…) I was having a class to go, so I was very furious.”

In general, students had received more support (from university staff) than others in attending the screening, which made it easier for them. Almost all, except a few students, preferred fixed appointments to drop in appointments. Many saw fixed appointments as necessary in order to make people understand that screening is mandatory and to “force” people to attend: “Fixed appointments are better, if not you will become lazy. I don’t have any spare time. With fixed time, you can take time away from work.” And: “It will be tricky if you can drop by at any time. It would take long time for me.. Tomorrow..? Or the day after tomorrow..?”

Many stressed that they were not used to communicate by letters and wanted reminders by phone. Several suggested to organise the screening as a one stop shop alongside other services, i.e. police verification.

## Discussion

### Health beliefs as motivation

The fact that many reported their perceptions of TB and the screening system as motivating factors for attending screening, can be elucidated by the health belief model (HBM) [13]. In order to explain health behaviour, HBM emphasises psychological factors such as rational thinking and expectations, rather than external conditions. According to the model, health behaviour is determined by people’s beliefs about a disease and about available strategies to avoid it [14]. The following are seen to influence whether a person will undertake a health action: perceived susceptibility to the disease, perceived severity of the disease, perceived benefits and barriers related to a health action, self-efficacy, and cues (“triggers”) to action. Most participants saw themselves as susceptible to TB, and believed TB to be a very severe disease. Most perceived screening to be beneficial in order to reduce the risk of TB. Perceived benefits were particularly related to getting the opportunity to undergo thorough technical examinations and procedures. This has been shown to be valued amongst some groups e.g. Somalis [15]. Barriers (inconvenient, time-consuming, unpleasant) were normally not considered to outweigh benefits. The letter served as a cue to action. Several mentioned phone reminders as helpful. Those who did not see themselves susceptible or did not see screening as beneficial (because of prior testing or vaccination) were less positive towards screening. Most saw themselves capable of accomplishing the screening (albeit with help of others). Those who had not undergone screening said they had believed they were not susceptible to TB, and named practical barriers and lack of self-efficacy as reasons for not attending.

Perceptions of weaknesses in the screening system can also be elucidated by HBM: Criticism of long time gaps between arriving in Norway and screening, and of absence of screening after return visits to high incidence countries, shows that participants were very concerned about the benefits of screening.

### Beyond beliefs: fear and stigma

HBM aspires to explain health behaviour by psychological factors; however, we find that certain significant psychological factors are not well captured by the model. Significant factors that emerged through interviews included fear and the feeling of stigmatisation. Being emotions, these may influence behaviour in a different manner than rational thoughts, which HBM focuses on. Fear was articulated by participants through expressions like “panic” and “freak out”. Fear differs from rational considerations of danger like “... if you don’t do anything about the illness today, you can be in danger tomorrow”, and fear is also shown to sometimes delay health actions [16]. Several mentioned how stigma affects people generally (hiding, unwilling to talk), without saying they felt stigmatised themselves. However, this does not necessarily mean they did not experience stigmatisation. According to Goffman’s classical works on stigma [17, 18], stigmatised people will always try to present themselves in such a way that the cause of stigma is hidden or less apparent. This is done by coping strategies to “supress and conceal any tendency to become shamefaced during encounters with others”. In order to reveal feelings of stigmatisation, we looked for stigma coping strategies and have identified the following:

#### Disidentifying

Disidentifying is a coping strategy about appearing different from the stigmatised. Talking negatively about others who are lazy and don’t attend screening, as many participants did, can be a way of disidentifying with them. The strategy implies validating a negative stereotype that one is afraid of being associated with, while at the same time resisting the application of this stereotype to oneself [19].

#### Compensating

Compensating is a well-recognised stigma coping strategy [20]. Important causes of TB stigma are that people with TB are perceived threatening because of infectiousness, and they are therefore held responsible and blamed for the disease [21, 22]. Thus; undergoing screening to detect and treat potential disease is a way of compensating. Participants underscored that they wanted to be responsible and take tests in order to protect others, which exemplifies this coping strategy.

#### Deshaming

The deshaming strategy entails attempts to alter the image of the stigmatised [23]. Being targeted by screening implies that one is regarded as a person who may have TB, which is stigmatising. A way of deshaming is to question this. An example is the participant who stressed that TB was not at all a problem in his country and claimed the reason behind screening was that TB had previously been a problem in Norway (i.e. the inclination to define diseases as “belonging to” other nations).

#### Denying

Denying can be a coping strategy [24], because by denying the existence of stigma, one eliminates the possibility of feeling stigmatised. Several participants denied having any knowledge of stigma related to TB or screening, or fear or discomfort related to testing. The participant who had never heard of anyone being afraid in the screening situation is an example. As fear and stigma related to TB is widely recognised [25], it seems unlikely that this was a completely new thought.

Although most saw screening as a good thing, fear and the feeling of stigmatisation created ambivalent attitudes. The participant who said stigma made her experience screening negatively, but that afterwards, with knowledge, thought of it as something positive, illustrates this. Ambivalence is also observed in other contexts where stigma is an issue. In a study about HIV in Burkina Faso, it is formulated this way:“… stigmatisation is a socially complex and ambiguous process (…) the same action can be perceived as either supportive or as stigmatising depending on the point of view, the point in time and the larger social situation in which it takes place.” [26] Accordingly, the question is not whether screening is stigmatising or not, but about the dynamics of stigma, and how an understanding of these dynamics can be used for adapting the system and reduce stigma as far as possible.

### Health literacy and physical accessibility

The fact that few understood the letter or knew how to act upon it without help of others, indicates low health literacy (HL). HL is a central topic in health system research and is seen as important for access to health care [27]. Initially, HL research focused on users’ abilities, and a commonly cited definition of HL is “The knowledge, skills and abilities that pertain to interactions with the healthcare system” [28]. Participants’ problems with language, finding the right transportation, and knowing whom to ask about screening, indicate their HL was low in the Norwegian context. HL can also be studied from a system perspective, something which has increasingly been done in recent years. In this perspective, a health-literate system is one that “makes it easier for people to navigate, understand and use information and services to take care of their health” [29]. That the letter was written in a language most participants did not understand, and few got information on how to access facilities, indicate weaknesses in the systems’ HL. The physical accessibility of the screening services, understood as the suitability of the location of services in relation to the location and mobility of users [30], was also perceived to be poor (difficulties with transport routes, finding facilities, getting through on the phone and distance to facilities).

We have seen that participants had problems with understanding the system and with physical access. Those who had not attended screening, reported these factors as reasons. Those who had attended dealt with the problems by asking others for help, and it sometimes made them feel vulnerable and embarrassed. The difficulties reveal a need for a more low-threshold arrangement of the system. As low-threshold arrangements make minimum demands on users, they are assumed to improve access, and have been recommended for TB case-finding among high risk groups in low burden settings [31].

### Autonomy and trust

Because screening is mandatory and participants were dependent on others to attend, it is important to examine how limitations in autonomy affect attitudes towards screening. Autonomy is a central topic in medical ethics [32, 33]. It can be defined as self-rule free from controlling interference and limitations, and autonomous persons are seen to act freely in accordance with a self-chosen plan [34]. Autonomy is regarded as a human need [35], and it is often assumed that coercive health measures damage patients’ trust in health systems [36, 37].

Discomfort due to dependence on others and feelings of coerciveness (e.g. screening felt like going to police) imply that lack of autonomy caused unease among participants. However, demand for consequences for non-attending indicates that many saw full autonomy in the screening process as detrimental. Both discomfort and demands for consequences differed between participant, and to explore possible reasons for these differences, we find self-determination theory (SDT) expedient [38]. SDT distinguishes between autonomous and controlled motivation: Autonomous motivation entails that behaviour is oriented towards self-endorsed interests and values, while controlled motivation entails that behaviour is oriented towards external directives about how to behave, think and feel. Following this, we define attending because of a wish to get treatment if sick as autonomously motivated, while attending because of “following rules”, consequences or reminders, as resulting from controlled motivation. A fixed appointment was preferred by most participants and entails a more controlled motivation than preferring drop in appointments.

There was a tendency for women and less educated people to be more driven by controlled motivation. According to SDT, controlled motivation results from a thwarting of the need for autonomy. Since women and less educated persons are subordinate in many settings [39], it seems likely that their autonomy has been thwarted. Davies and Elwyn [40] make a similar point, holding that people in less powerful groups are limited in their autonomy because they lack resources to choose freely between alternative actions. Many participants expressed a lack of resources (time, capacity, understanding of the system) to think about their health and plan screening attendance, and therefore found “external directives” like reminders and fixed appointments helpful. Davies and Elwyn argue that to enhance equity in health care, one should address limitations in autonomy of disadvantaged groups before “over-promoting autonomy” in general. In our case, addressing limitations in autonomy would entail measures to make it easier to understand the system and attend without help of others.

Concerning coercion, we understand that although coerciveness caused discomfort amongst participants, it was not perceived as the most problematic element of screening, or the most crucial for trust. Statements indicate that feelings of trust and safety were even more dependent on whether they found the screening system comprehensible, competent and useful, that they understood why they were singled out for screening, and that they received test results. Most saw “external directives” as help to attend and expressed that they found this help more important than autonomy in the process. That women and less educated underlined this most clearly, concurs with observations that people in subordinate positions tend to develop behaviour of contentment and gratitude [41–43].

### Ambivalence and its implications

Although participants generally were positive towards the principle of screening, fear, stigma, and practical problems created negative attitudes. It is vital to recognise that just as different people have different attitudes, an individual often has multiple and contradictory attitudes simultaneously, and hence is ambivalent. To build trust and enhance screening uptake, one should take both negative and positive attitudes into account.

Because ambivalence makes it difficult for people to make decisions about health [44, 45], it is important to reduce it as far as possible. Communication with users should target reducing ambivalence [46] by emphasising aspects that are seen as positive, and “respond to” aspects that are seen as negative. The former can be done by underlining the positive health purposes of the screening, the latter by highlighting that screening is routinely conducted on all people from high TB incidence areas, and that TB is curable and treatment is free of cost. Since people appreciate both friendliness and firmness and are motivated by obligation, one should endeavour to communicate in a way that is both respectful and friendly, and at the same time clear and firm.

Since ambivalence causes difficulties in decision-making, optional screening may lead to more people postponing or not attending. Hence, it seems reasonable that screening is kept mandatory. However as this implies a thwarting of autonomy, which is a need that should be satisfied as far as possible, lack of autonomy should be compensated by empowering users. Empowering measures include providing information in a language users understand, ensuring physical accessibility and providing explanations on how to reach services, and meeting users in a respectful and friendly way.

## Limitations

Only three out of 34 participants had not attended screening; hence we got limited explicit information about reasons for not attending. Most of the informants had come for family reunification, and we therefore got less information about the situation of students and work migrants. The fact that interviews and focus group discussions were not conducted in the participants’ native languages could affect their opportunity to elaborate on answers. However, we experienced that participants took active part in the discussion despite their sometimes limited vocabulary. We made efforts to follow up on questions and responses when necessary, in order to avoid misunderstandings and get as much information as possible.

## Conclusions

We have seen that both beliefs, emotions and practical and situational aspects influenced participants’ experiences and attitudes towards screening. This complexity, including stigma, the severity of TB, coerciveness in screening, and being new in the country, made participants ambivalent and vulnerable. In order to meet users’ needs, enhance trust and uptake of screening, all aspects of this complexity should be taken into account. Low-threshold arrangements should be promoted. In this context, low-threshold entails easy access to facilities and to information in a language users understand. It does not entail freedom from external directives; since informants were ambivalent and vulnerable, they needed external directives as support to accomplish screening. Hence, the screening services should be easily accessible and friendly, but with structures to “supervise” and follow up.

We conclude that in a Norwegian context, mandatory screening seems justifiable and reasonable, but that empowering measures to counteract its potential negative effects should be taken. Also, it is our view that mandatory screening creates a distinct obligation for the health system to follow up after screening, by providing all screening results and comprehensive information.

## Additional files

Additional file 1: Population characteristics (PDF 5 kb)

Additional file 2: Interview guide (PDF 179 kb)

Additional file 3: COREQ checklist (PDF 123 kb)

## Data Availability

The datasets used and/or analysed during the current study are available from the corresponding author on reasonable request.

## Abbreviations

HBM: Health Belief Model
HL: Health Literacy
SDT: Self-Determination Theory
TB: Tuberculosis
