# Supplementary material for: Wanting to or having to – a qualitative study of experiences and attitudes towards migrant screening for tuberculosis in Norway
Source: BMC Public Health. 2019 Jun 21;19:796. doi: 10.1186/s12889-019-7128-z (PMC6588894; doi:10.1186/s12889-019-7128-z)
Supplement: Supplementary file 1 — Population characteristics (PDF 5 kb) [file 12889_2019_7128_MOESM1_ESM.pdf]

Additional file 1: Population characteristics

Country of origin:

Africa (23): Congo (1), Eritrea (6), Ethiopia (5), Ghana (2), Nigeria (1), Senegal (1), Somalia (5), Tunisia (1), Uganda (1).
Asia (9): Burma (1), India (3), Iraq (1), Pakistan (1), Philippines (3).
Europe (2): Ukraine, Turkey.

Language spoken:

Burma: Norwegian (1).
Congo: Norwegian (1).
Eritrea: Norwegian (5), English (1).
Ethiopia: Norwegian (2), English (3).
Ghana: English (2).
India: Norwegian (2), English (1).
Iraq: Norwegian (1).
Nigeria: English (1).
Pakistan: Norwegian (1).
Philippines: Norwegian (3).
Senegal: Norwegian (1).
Somalia: Norwegian (3), translator to Norwegian (2).
Tunisia: Norwegian (1).
Turkey: Norwegian (1).
Uganda: Norwegian (1).
Ukraine: Norwegian (1).
