# Supplementary material for: Wanting to or having to – a qualitative study of experiences and attitudes towards migrant screening for tuberculosis in Norway
Source: BMC Public Health. 2019 Jun 21;19:796. doi: 10.1186/s12889-019-7128-z (PMC6588894; doi:10.1186/s12889-019-7128-z)
Supplement: Supplementary file 2 — Interview guide (PDF 179 kb) [file 12889_2019_7128_MOESM2_ESM.pdf]

Additional file 2 - Interview guide (questions and follow-up questions)

- How did you learn about the tuberculosis screening the first time?
- Did you receive an invitation letter? Can you tell us a little about the content of the letter? What do you think about the information in the letter?
- How did you make an appointment? What do you think about having a fixed appointment? What do you think about drop in appointments?
- How was it to find the facility where the examinations were to be taken?
- Can you please tell us a little about what information you were given during the examinations? What do you think about this information?
- How did you experience the examinations?
- Did you receive the results of the examinations? How were the results presented?
- How did you perceive the information you were given about the results of the examinations?
- Could you please tell us a little about what made you attend/not attend the screening?
- Did you have any particular positive or negative experiences related to the examinations? If you had negative experiences, do you have any suggestions on how to do it differently?
- Do you have any suggestions on how to improve the organisation of the screening?
