# Supplementary material for: Wanting to or having to – a qualitative study of experiences and attitudes towards migrant screening for tuberculosis in Norway
Source: BMC Public Health. 2019 Jun 21;19:796. doi: 10.1186/s12889-019-7128-z (PMC6588894; doi:10.1186/s12889-019-7128-z)
Supplement: Supplementary file 3 — COREQ checklist (PDF 123 kb) [file 12889_2019_7128_MOESM3_ESM.pdf]

Additional file 3: COREQ checklist

Domain 1: Research team and reflexivity

Personal Characteristics

1. Interviewer/facilitator Which author/s conducted the interview or focus group? IN and MD.

2. Credentials What were the researcher's credentials? E.g. PhD, MD. IN: cand polit., MD: cand. polit., TWS: MD PhD, BAW: MPH PhD.

3. Occupation What was their occupation at the time of the study? IN: Advisor, LHL International Tuberculosis Foundation; MD: Deputy director, LHL International Tuberculosis Foundation; TWS: Public Health Specialist, Health Agency, City of Oslo. BAW: Senior Researcher, Norwegian Institute of Public Health.

4. Gender Was the researcher male or female? IN, MD, BAW: female, TWS: male.

5. Experience and training What experience or training did the researcher have? IN and MD are social anthropologists and have long term experience with qualitative research methods and field work. They have been working for many years with outreach information- and support activities related to TB, targeting immigrant groups most affected by TB in Norway. TWS and BAW have long-tern experience with TB research within epidemiology and operational research in the Norwegian context.

Relationship with participants

6. Relationship established Was a relationship established prior to study commencement? We had been cooperating in a previous project with one of the participants. With the others, there were no prior relationship.

7. Participant knowledge of the interviewer What did the participants know about the researcher? e.g. personal goals, reasons for doing the research. We explained that our organisation works to combat tuberculosis and that this research was conducted to understand users' experiences with the screening system in order to adapt the screening system.

8. Interviewer characteristics What characteristics were reported about the interviewer/facilitator? e.g. Bias, assumptions, reasons and interests in the research topic. We explained that our organisation works to combat tuberculosis globally and in Norway and that the aim of this research was to understand users' experiences with the screening system in order to adapt the screening system. User-involvement is important for increased uptake of screening programmes.

Domain 2: study design

Theoretical framework

9. Methodological orientation and Theory

What methodological orientation was stated to underpin the study? e.g. grounded theory, discourse analysis, ethnography, phenomenology, content analysis. A general inductive approach was used.

Participant selection

10. Sampling How were participants selected? e.g. purposive, convenience, consecutive, snowball. We used both purposive, convenience and snowball sampling.

11. Method of approach How were participants approached? e.g. face-to-face, telephone, mail, email Participants were approached by email contact or face-to-face inquiry from IN/MD, telephones or face-to-face inquiry from INs/MDs contacts in the immigrant population and Oslo Adult education.

12. Sample size How many participants were in the study? 34.

13. Non-participation How many people refused to participate or dropped out? Reasons? Unfortunately, the number that were invited, but not enrolled, was not recorded. Those who refused

said they did not have the opportunity to participate, or similar reasons. No people dropped out after enrollment.

Setting

14. Setting of data collection Where was the data collected? e.g. home, clinic, workplace. At the researchers office, at Oslo Adult Education and at a café (one individual interview). The participants were able to choose the place of the interview/focus group discussion.

15. Presence of non-participants Was anyone else present besides the participants and researchers? No.

16. Description of sample What are the important characteristics of the sample? e.g. demographic data, date. The participants came from 16 different countries in Africa (23), Asia (9) and Europe (2). 23 were women and 11were men. 23 came for family reunification, three were au pairs, two were work migrants, and six were students.

Data collection

17. Interview guide Were questions, prompts, guides provided by the authors? Yes. Was it pilot tested? No.

18. Repeat interviews Were repeat interviews carried out? No. If yes, how many?

19. Audio/visual recording Did the research use audio or visual recording to collect the data? Yes.

20. Field notes Were field notes made during and/or after the interview or focus group? No.

21. Duration What was the duration of the interviews or focus group? 45 to 94 minutes.

22. Data saturation Was data saturation discussed? Yes.

23. Transcripts returned Were transcripts returned to participants for comment and/or correction? No.

Domain 3: analysis and findings

Data analysis

24. Number of data coders How many data coders coded the data? Two.

25. Description of the coding tree Did authors provide a description of the coding tree? No.

26. Derivation of themes Were themes identified in advance or derived from the data? Derived from the data.

27. Software What software, if applicable, was used to manage the data? Software was not used.

28. Participant checking Did participants provide feedback on the findings? No.

Reporting

29. Quotations presented Were participant quotations presented to illustrate the themes / findings? Yes. Was each quotation identified? e.g. participant number No.

30. Data and findings consistent Was there consistency between the data presented and the findings? Yes.

31. Clarity of major themes Were major themes clearly presented in the findings? Yes.

32. Clarity of minor themes Is there a description of diverse cases or discussion of minor themes? Yes.
